# Efficacy of multimodal analgesia based on the concept of enhanced recovery after surgery in laparoscopic radical gastrectomy for gastric cancer

**DOI:** 10.12669/pjms.40.10.10088

**Published:** 2024-11

**Authors:** Lingli Xu, Lu Yao, Jianfen Qin, Hongzhen Xu

**Affiliations:** 1 Lingli Xu, Nursing Department, Sir Run Run Shaw Hospital, Zhejiang University School of Medicine, Hangzhou, Zhejiang Province 310016, P.R. China; 2 Lu Yao, Nursing Department, Sir Run Run Shaw Hospital, Zhejiang University School of Medicine, Hangzhou, Zhejiang Province 310016, P.R. China; 3 Jianfen Qin, Nursing Department, Sir Run Run Shaw Hospital, Zhejiang University School of Medicine, Hangzhou, Zhejiang Province 310016, P.R. China; 4 Hongzhen Xu Department of Nursing, The Children’s Hospital Zhejiang University School of Medicine Hangzhou, Hangzhou City, Zhejiang Province 310052, P.R. China

**Keywords:** Enhanced recovery after surgery, Multimodal analgesia, Laparoscopic radical gastrectomy, Gastric cancer

## Abstract

**Objective:**

To explore the effect of multimodal analgesia based on the concept of enhanced recovery after surgery (ERAS) in patients undergoing laparoscopic radical gastrectomy (LRG) for gastric cancer (GC).

**Methods:**

Clinical data of 128 patients undergoing LRG for GC, admitted to Sir Run Run Shaw Hospital, Zhejiang University School of Medicine from March 2021 to March 2022, were retrospectively analyzed. Among them, 66 patients received a multimodal analgesic management based on ERAS (ERAS group), and 62 patients were treated with conventional mode of analgesia (control group). Pain levels, rehabilitation status, as well as inflammatory factors and stress response indicators before and after surgery were compared between the two groups.

**Results:**

There was no significant difference in baseline data between the two groups (*P*>0.05). The postoperative pain and recovery in the ERAS group were better than those in the control group (*P*<0.05). After the surgery, serum levels of tumor necrosis factor-alpha (TNF-α), Interleukin 6 (IL-6), and C-reactive protein (CRP) in both groups increased compared to before the surgery, but were significantly lower in the ERAS group compared to the control group (*P*<0.05). After the surgery, serum malondialdehyde (MDA) and xanthine oxidase (XOD) levels in both groups increased, while superoxide dismutase (SOD) levels decreased compared to preoperative levels. The observed postoperative levels of serum MDA and XOD were significantly lower in the ERAS group, while the postoperative SOD levels were higher compared to the control group (*P*<0.05).

**Conclusions:**

Patients undergoing LRG for GC can benefit from a multimodal pain management plan based on ERAS to reduce postoperative pain, alleviate inflammation, stress responses, and shorten the postoperative recovery process.

## INTRODUCTION

Gastric cancer (GC) is the fifth most common cancer worldwide, accounting for 4.9% of all cancers globally.^1^,^2^ In 2022, age-standardized (world) incidence rate of GC was 9.2 per 100,000 population.^1^ Surgical resection remains the main treatment modality in cases of GC, especially at early stages of the disease.^2^,^3^ Recently, less invasive laparoscopic radical gastrectomy (LRG) for GC resection has become increasingly popular, with good results and high safety.^3^,^4^ However, LRG for GC is still an invasive method that can trigger stress responses, leading to varying degrees of postoperative pain and adverse effects on the overall recovery.^5^,^6^

Therefore, effective pain management in GC patients after LRG is crucial. Opioids are commonly used for postoperative analgesia, but are associated with the increased risk of adverse reactions such as respiratory depression, intestinal obstruction, urinary retention, vomiting and nausea, which may prolong postoperative rehabilitation and hospital stay.^7^,^8^ Multimodal analgesia refers to the combination of analgesic methods and drugs with different mechanisms of action to fully exert analgesic effects and reduce the occurrence of adverse reactions.^9^

The concept of enhanced recovery after surgery (ERAS) involves implementing interventions based on evidence-based medicine to alleviate surgical stress and pain to reduce the risk of complications, and shorten the postoperative recovery process.^10^,^11^ It has been demonstrated that ERAS is beneficial for patients undergoing either laparoscopic-assisted or robotic gastrectomy for gastric cancer,^12^ and multimodal analgesia has also been reported to be an essential part of ERAS.^13^ However, few studies have explored the effect of multimodal analgesia based on the concept of ERAS in patients undergoing LRG for GC. This retrospective study included clinical data of 128 patients who underwent LRG for GC to clarify the intervention value of a multimodal pain management plan based on ERAS.

## METHODS

Clinical data of patients who undergoing LRG for GC, admitted to Sir Run Run Shaw Hospital, Zhejiang University School of Medicine from March 2021 to March 2022, were selected.

### Inclusion & Exclusion Criteria:

Patients diagnosed with GC by pathological examination^14^ and underwent LRG for GC with complete clinical data were included. Patients with mental illness, history of alcohol and drug dependence, transferred to open surgery, complicated with other malignant tumors, or received analgesic medication treatment within one week before surgery were excluded. Patients treated using conventional analgesic methods were assigned to the control group, while patients received a multimodal pain management plan based on ERAS were assigned to the ERAS group.

### Ethical Approval:

The ethics committee of our hospital approved this study with the 2022-460-01 on May 23, 2022.

### Routine pain management:

Basic information on LRG, anesthesia, and pain management were explained to the patient in detail preoperatively. Patients also received dietary guidance. Patients were routinely monitored for electrocardiogram, blood pressure, and blood oxygen saturation after entering the operating room. Routine anesthesia induction was conducted with 0.02 mg/kg midazolam (manufactor: Jiangsu nhwa Pharmaceutical Group Co., Ltd., China), 0.3 μg/kg sufentanil (manufactor: Yichang Humanwell Pharmaceutical Co., Ltd, China), 0.2-0.3 mg/kg etomidate (manufactor: Jiangsu nhwa Pharmaceutical Group Co., Ltd., China), and 0.3 mg/kg cisatracurium (manufactor: Zhejiang Xianju Pharmaceutical Co., Ltd, China). Then mechanical ventilation was started after tracheal intubation. Intraoperative anesthesia was maintained with 0.1-0.3 μg/(kg·min) remifentanil (manufactor: China National Pharmaceutical Industrial Corporation Ltd. Langfang Branch, China) and 3-6mg/(kg·h) propofol. (manufactor: Xi’an Libang Pharmaceutical Co., Ltd, China) Patients were maintained at a tidal volume of 8-10 ml/kg, respiratory rate of 12-14 breaths/minute, and end-tidal carbon dioxide of 35-45 mmHg. Patient-controlled intravenous analgesia (PCIA) pump with 2 μg/kg sufentanil diluted to 100mL with saline was used for postoperative pain relief. The parameters for PCIA were as follows: loading dose, 2 mL; background infusion dose, 2 mL/h; and lockout time, 15 minutes. When required, instructions and guidance for in-bed exercise.

### Multimodal pain management based on ERAS:

The multimode pain management is as follows:

- Pain intervention team, comprised of one surgeon, two responsible nurses, one anesthesiologist, one pain specialist nurse, and one pharmacist.
- Preoperative preparation: Distribution of relevant health knowledge brochures to patients, explaining the ERAS concept and perioperative pain relief measures to assist patients to fully understand perioperative pain and related intervention measures. Preoperative guidance, including starting semi liquid diet one day before the surgery, drinking 750 ml of glucose water 10 hours and two hours before the surgery, respectively, fastin
- Maintaining body temperature of patients during the surgery: covering patient’s non-essential exposed areas, provide insulation tr

### Multimode analgesia management:

Patients received advanced pain management with 40 mg parecoxib sodium (manufactor: Pfizer, USA) intravenously injected 30 minutes before anesthesia induction; Intraoperative administration of 0.5% ropivacaine (manufactor: Qilu Pharmaceutical Co., Ltd, China) for incision and subcutaneous tissue infiltration; Timely discharge of carbon dioxide gas from the abdominal cavity after the surgery, and cleaning the abdominal ca

### Postoperative measures:

after return to the ward, informing patients of common postoperative complications and precautions; Regularly inquiring about the patient’s subjective feelings, discomfort, and degree of pain; Adjusting pain relief measures in a timely manner based on patient complaints and medical advice; 12 hours after the surgery, assisting patients in simple activities such as turning over and sitting in bed, and encouraging patients to stand and get out of bed; Ensuring postoperative normal drinking water, no intake of any food within 12 hours; Continuously replenishing fluids on the 1st day after the surgery and guiding the patient to take oral nutrient solution; Subsequently, based on the patient’s physical condition, guiding them to abstain from liquid and semi liquid foods, as well as regular foods.

### Observation indicators:

- Pain levels including postoperative pain severity at different times (four hours, 12 hours, 24 hours, and 48 hours), number of presses on the analgesic pump, and number of additional analgesics. The degree of pain was evaluated using the visual analogue scale (VAS) with a total score of 10 points.^15^ Higher the score indicated stronger pain.
- Rehabilitation status including time to first exhaust, time to first defecation, time to first getting out of bed, and length of hospital stay.
- Inflammatory factor indicators including levels of tumor necrosis factor-α (TNF-α), interleukin-6 (IL-6), and C-reactive protein (CRP). Levels of inflammatory factors were detected in the serum from 4 ml of fasting venous blood by enzyme-linked immunosorbent assay.
- Stress response indicators including levels of serum malondialdehyde (MDA), xanthine oxidase (XOD), and superoxide dismutase (SOD) were measured in the serum from 4 ml of fasting venous blood by enzyme-linked immunosorbent assay.

### Statistical Analysis:

All data analyses were conducted using SPSS 25.0 software (IBM Corp, Armonk, NY, USA). Quantitative data were represented by mean ± standard deviation, and student’s *t*-test was used for intergroup comparison, and paired *t*-test was used for intragroup comparison before and after the surgery. Count data were represented by the number of cases, and analyzed using Chi-square test. *P*<0.05 indicated statistically significant difference.

## RESULTS

A total of 128 GC patients (73 males and 55 females) who underwent LRG met the eligibility criteria were included in this retrospective study. Age of the patients ranged from 38 to 79 years, with a mean of 60.33 ± 10.54 years. Among them, 62 patients received conventional method of analgesia, and 66 patients received multimodal analgesic management plan based on ERAS. There was no significant difference in baseline data between the two groups (*P*>0.05) (Table-I).

**Table-I:** Comparison of baseline data between two groups.

Group	Gender (male/female)	Age (year)	BMI (kg/m^2^)	ASA staging		

				I	II	III
Control group (n=62)	38/24	59.77±9.84	23.15±256	7 (11.29)	29 (46.77)	26 (41.94)
ERAS group (n=66)	35/31	60.85±11.21	22.81±2.99	11 (16.67)	31 (46.97)	24 (36.36)
*χ*^2^/*t*	0.890	-0.575	0.698	0.911		
*P*	0.345	0.567	0.487	0.634		

BMI: body mass index, ASA staging: American Society of Anesthesiologists staging.

The VAS score of the ERAS group was significantly lower than that of the control group at four, 12, 24, and 48 hours after the surgery. Similarly, the number of compressions of the analgesic pump and the number of additional analgesics in the ERAS group were significantly lower than in the control group (*P*<0.05) (Table-II).

**Table-II:** Comparison of pain levels between two groups.

Group	VAS score				Number of compressions of analgesic pump (times)	Number of additional analgesics (times)

	4-hours after surgery	12-hours after surgery	24-hours after surgery	48-hours after surgery		
Control group (n=62)	4.05±0.93	3.75±0.78	3.50±0.67	2.85±0.52	10.31±2.44	3.96±1.11
ERAS group (n=66)	2.86±0.53	2.58±0.46	2.32±0.39	1.98±0.28	7.24±1.95	2.21±0.81
*t*	8.962	10.412	12.267	11.883	7.887	10.233
*P*	<0.001	<0.001	<0.001	<0.001	<0.001	<0.001

VAS: visual analogue scale.

The ERAS group had significantly shorter time to first exhaust, first bowel movement, first time out of bed, and shorter hospital stay compared to the control group (*P*<0.05) (Table-III). Before the surgery, there was no significant difference in serum TNF-α, IL-6, and CRP levels between the two groups (*P*>0.05). After the surgery, serum concentrations of TNF-α, IL-6, and CRP in both groups significantly increased compared to preoperative levels, but were overall significantly lower in the ERAS group compared to the control group (*P*<0.05) (Table-IV).

**Table-III:** Comparison of Rehabilitation Status between Two groups.

Group	First exhaust time (hour)	First bowel movement time (day)	First time getting out of bed (day)	Hospitalization duration (day)
Control group (n=62)	34.73±5.69	4.09±1.14	3.52±1.09	7.41±1.23
ERAS group (n=66)	20.47±3.35	3.05±1.06	2.31±0.98	5.70±1.13
*t*	17.403	5.348	6.612	8.197
*P*	<0.001	<0.001	<0.001	<0.001

**Table-IV:** Comparison of levels of inflammatory factor indicators between two groups.

Time	Group	TNF-α (ng/L)	IL-6 (pg/ml)	CRP (mg/L)
Before surgery	Control group (n=62)	64.46±9.33	2.64±0.68	13.21±2.46
	ERAS group (n=66)	63.65±8.32	2.59±0.71	12.65±2.39
	*t*	0.519	0.406	1.306
	*P*	0.605	0.685	0.194
After surgery	Control group (n=62)	99.14±9.35^a^	21.64±5.62^a^	28.89±6.05^a^
	ERAS group (n=66)	86.72±6.76^a^	15.75±3.79^a^	21.27±4.71^a^
	*t*	8.651	6.990	7.978
	*P*	<0.001	<0.001	<0.001

a P<0.05, compared with the same group before surgery; TNF-α: tumor necrosis factor-α; IL-6: interleukin-6; CRP: C-reactive protein.

Before the surgery, there was no significant difference in serum MDA, SOD, and XOD levels between the two groups (*P*>0.05). After the surgery, serum MDA and XOD levels in both groups were significantly increased compared to preoperative levels, while SOD levels were significantly reduced. However, serum MDA and XOD levels in the ERAS group were significantly lower, while the SOD levels were significantly higher than those in the control group (*P*<0.05) (Table-V).

**Table-V:** Comparison of stress response index levels between two groups.

Time	Group	MDA (mmol/L)	SOD (U/ml)	XOD (U/ml)
Before surgery	Control group (n=62)	5.01±1.06	94.05±10.96	5.58±1.51
	ERAS group (n=66)	4.86±1.19	92.82±11.04	5.72±1.35
	*t*	0.751	0.632	0.554
	*P*	0.454	0.528	0.581
After surgery	Control group (n=62)	7.68±1.45^a^	61.44±8.21^a^	10.26±3.45^a^
	ERAS group (n=66)	5.97±1.07^a^	70.43±9.35^a^	8.19±2.24^a^
	*t*	7.623	5.765	4.050
	*P*	<0.001	<0.001	<0.001

a P<0.05, compared with the same group before surgery, MDA: malondialdehyde, XOD: xanthine oxidase, SOD: superoxide dismutase.

## DISCUSSION

The results of this study show that the multimodal pain management scheme based on ERAS has high application value in patients undergoing LRG for GC. It efficiently alleviated postoperative pain and shortened the time for postoperative functional recovery. Simpson et al.^16^ has pointed out that the comprehensive application of different analgesic methods and drugs, through synergistic effects, can effectively reduce the degree of pain, alleviate the level of micro inflammation and stress response, and ensure good outcomes of the disease. Similarly, our results showed that multimodal pain management plan based on ERAS resulted in better outcomes compared to standard mode of anesthesia. We may speculate that the preoperative preemptive analgesia that is a part of the multimodal pain management can prevent postoperative pain hypersensitivity.

Additionally, postoperative patient-controlled epidural analgesia can suppress pain severity through continuous medication, alleviate patient pain, ensure early oral feeding and in-bed movement, and thus reduce the risk of complications. Gustafsson et al.^17^ and Cao et al.^18^ studied the use of a multimodal pain management approach based on the ERAS concept in patients undergoing colorectal cancer surgery, and confirmed that it can reduce postoperative pain and is of great significance in reducing the risk of complications. Their research also indicated that postoperative epidural analgesia can alleviate the degree of neuroendocrine stress response, ensure postoperative analgesic effect, promote gastrointestinal activity, and accelerate postoperative recovery.

Kang et al.^19^ adopted a management plan based on the ERAS concept to intervene in laparoscopic GC patients, and showed that the ERAS group had shorter postoperative rehabilitation time, with lower pain severity scores compare to the control group 1-4 days after the surgery. Together with our observations, these results confirm that the management model based on the ERAS concept can achieve superior analgesic effect by blocking the pain mechanism at different time points and targets through anesthesia regimens and analgesics with different mechanisms of action, inhibiting central and peripheral sensitization. It is plausible that since conventional analgesic measures are based on relatively limited number of agents, they cannot achieve the required reduction in the degree of pain and are unable to alleviate the degree of inflammatory stress response.^20^ On the other hand, multimodal pain management scheme based on ERAS can reduce postoperative pain and stress response levels through multidisciplinary collaboration and optimization of perioperative interventions.^16^-^19^

The results of this study showed that the serum levels of TNF-α, IL-6, and CRP in the ERAS group after surgery were lower. Tissue damage can have a stimulating effect on the body, promoting the release of inflammatory mediators and causing pain sensitization,^21^-^23^ and numerous factors participate in inflammation and pain responses. IL-6, a cytokine, generated by macrophages and neutrophils, is closely related to inflammation and pain severity.^21^ CRP is produced during tissue damage, and its levels positively correlate with the occurrence and progression of inflammatory responses.^22^ TNF-α is the earliest inflammatory mediator in the inflammatory response process, and can promote the release and synthesis of other cytokines.^23^

The study also showed that serum levels of MDA, and XOD in the ERAS group after surgery were lower, while the SOD levels were higher than those in the control group (*P*<0.05). MDA, SOD, and XOD are common clinical indicators for evaluating the degree of stress response that is also an important indicator of the degree of trauma.^24^ Our results, therefore, further confirm that the multimodal analgesic management scheme based on ERAS can reduce the degree of inflammation and stress response, and thus, has a high clinical value. We may speculate that this method of anesthesia promotes the stability of the internal environment. Due to its multimodal nature, it can simultaneously act on different targets or phases of the physiological and pathological mechanisms of pain, increase the pain threshold, alleviate the degree of stress response and micro inflammatory state. Moreover, multimodal analgesic management scheme based on ERAS can prevent central and peripheral sensitization of pain, reducing overall sensitivity to pain.^25^-^27^

### Limitations:

Firstly, this is a single center retrospective study with small sample size, which may lead to selection bias. Secondly, neither group was randomly assigned. Therefore, baseline information may have been imbalanced and biased. Thirdly, the follow-up period for postoperative pain efficacy was only 48 hours. Future studies with longer follow-up times are needed to confirm the results, and to analyze the impact of both methods on the long-term functional recovery of patients. Further high-quality research is needed to validate our observations.

## CONCLUSION

Patients undergoing LRG for GC can benefit from a multimodal pain management plan based on ERAS to reduce postoperative pain, alleviate inflammation and stress responses, and shorten the duration of postoperative recovery.

### Authors’ contributions:

**LX:** Conceived and designed the study.

**LY**, **JQ** and **HX:** Collected the data, performed the analysis and Review.

**LX:** Was involved in the writing of the manuscript and is responsible for the integrity of the study.

All authors have read and approved the final manuscript.

## References

[1] Global Cancer Observatory:Cancer Today Lyon, France:International Agency for Research on Cancer.

[2] Bray F, Laversanne M, Sung H, Ferlay J, Siegel RL, Soerjomataram I (2024). Global cancer statistics 2022:GLOBOCAN estimates of incidence and mortality worldwide for 36 cancers in 185 countries. CA Cancer J Clin.

[3] Tian Y, Cao S, Li L, He Q, Xia L, Jiang L (2020). Effects of perioperative enhanced recovery after surgery pathway management versus traditional management on the clinical outcomes of laparoscopic-assisted radical resection of distal gastric cancer:study protocol for a randomized controlled trial. Trials.

[4] Alakus H, Kaya M, Ozer H, Egilmez HR, Karadayi K (2021). ADAM10 expression in gastric adenocarcinoma:Results of a curative gastrectomy cohort. Pak J Med Sci.

[5] Li MZ, Wu WH, Li L, Zhou XF, Zhu HL, Li JF (2018). Is ERAS effective and safe in laparoscopic gastrectomy for gastric carcinoma?A meta-analysis. World J Surg Oncol.

[6] Hu Y, Zhao G, Zheng H (2015). Therapeutic effects of laparotomy and laparoscopic surgery on patients with gastric cancer. Pak J Med Sci.

[7] Wang L, Wu LX, Han Z, Ma WH, Geng ZH (2022). Effect of flurbiprofen axetil combined with “Cocktail”therapy on opioid dosage in patients after total knee arthroplasty. Pak J Med Sci.

[8] Wang W, Ma Y, Liu Y, Wang P, Liu Y (2022). Effects of Dexmedetomidine Anesthesia on Early Postoperative Cognitive Dysfunction in Elderly Patients. ACS Chem Neurosci.

[9] Zhu Z, Chen B, Ye W, Wang S, Xu G, Pan Z (2019). Clinical significance of wound infiltration with ropivacaine for elderly patients in china underwent total laparoscopic radical gastrectomy:A retrospective cohort study. Medicine (Baltimore).

[10] Zhao X, Chen L, Huang F, Huang Z, Zhou H (2023). Enhanced Recovery after Surgery in patients undergoing total joint arthroplasty:A retrospective study. Pak J Med Sci.

[11] Yang FZ, Wang H, Wang DS, Niu ZJ, Li SK, Zhang J (2020). The effect of perioperative ERAS pathway management on short-and long-term outcomes of gastric cancer patients. Zhonghua Yi Xue Za Zhi.

[12] Wang Y, Luo S, Wang S (2023). Evaluation of enhanced recovery after surgery for gastric cancer patients undergoing gastrectomy:a systematic review and meta-analysis. Wideochir Inne Tech Maloinwazyjne.

[13] Beverly A, Kaye AD, Ljungqvist O, Urman RD (2017). Essential Elements of Multimodal Analgesia in Enhanced Recovery After Surgery (ERAS) Guidelines. Anesthesiol Clin.

[14] Health Commission of The People's Republic Of China N (2022). National guidelines for diagnosis and treatment of gastric cancer 2022 in China (English version). Chin J Cancer Res.

[15] Price DD, McGrath PA, Rafii A, Buckingham B (1983). The validation of visual analogue scales as ratio scale measures for chronic and experimental pain. Pain.

[16] Simpson JC, Bao X, Agarwala A (2019). Pain Management in Enhanced Recovery after Surgery (ERAS) Protocols. Clin Colon Rectal Surg.

[17] Gustafsson UO, Scott MJ, Hubner M, Nygren J, Demartines N, Francis N (2019). Guidelines for Perioperative Care in Elective Colorectal Surgery:Enhanced Recovery After Surgery (ERAS®) Society Recommendations:2018. World J Surg.

[18] Cao L, Zhang L, Chen B, Yan L, Shi X, Tian L (2024). Application of multimodal standardized analgesia under the concept of enhanced recovery after surgery in laparoscopic radical colorectal cancer surgery. Front Oncol.

[19] Kang SH, Lee Y, Min SH, Park YS, Ahn SH, Park DJ (2018). Multimodal Enhanced Recovery After Surgery (ERAS) Program is the Optimal Perioperative Care in Patients Undergoing Totally Laparoscopic Distal Gastrectomy for Gastric Cancer:A Prospective, Randomized, Clinical Trial. Ann Surg Oncol.

[20] Liao YQ, Min J, Wu ZX, Hu Z (2023). Comparison of the effects of remimazolam and dexmedetomidine on early postoperative cognitive function in elderly patients with gastric cancer. Front Aging Neurosci.

[21] Sato B, Kanda M, Tanaka C, Kobayashi D, Iwata N, Hattori N (2018). Significance of Preoperative Systemic Inflammation Score in Short-Term and Long-Term Outcomes of Patients with Pathological T2-4 Gastric Cancer After Radical Gastrectomy. World J Surg.

[22] Lu J, Xu BB, Xue Z, Xie JW, Zheng CH, Huang CM (2021). Perioperative CRP:A novel inflammation-based classification in gastric cancer for recurrence and chemotherapy benefit. Cancer Med.

[23] Shoka M, Kanda M, Ito S, Mochizuki Y, Teramoto H, Ishigure K (2020). Systemic Inflammation Score as a Predictor of Pneumonia after Radical Resection of Gastric Cancer:Analysis of a Multi-Institutional Dataset. Dig Surg.

[24] Chen C, Fu YH, Li MH, Ruan LY, Xu H, Chen JF (2019). Nuclear magnetic resonance-based metabolomics approach to evaluate preventive and therapeutic effects of Gastrodia elata Blume on chronic atrophic gastritis. J Pharm Biomed Anal.

[25] Tian Y, Lin Y, Sun C, Lowe S, Bentley R, Yang P (2023). Comparison of short-term efficacy and safety between total robotic and total 3D laparoscopic distal radical gastrectomy for gastric cancer in Enhanced Recovery After Surgery (ERAS) protocol:a propensity score matching study. J Robot Surg.

[26] Tian Y, Li Q, Pan Y (2022). Prospective study of the effect of ERAS on postoperative recovery and complications in patients with gastric cancer. Cancer Biol Med.

[27] Wang J, Luo Y, Wang Q, Bai J, Liao Q, Feng X (2020). Evaluation of the application of laparoscopy in enhanced recovery after surgery (ERAS) for gastric cancer:a Chinese multicenter analysis. Ann Transl Med.

